# A Localized Coverage Preserving Protocol for Wireless Sensor Networks

**DOI:** 10.3390/s90100281

**Published:** 2009-01-08

**Authors:** Yuheng Liu, Juhua Pu, Shuo Zhang, Yunlu Liu, Zhang Xiong

**Affiliations:** School of Computer Science and Engineering, Beihang University, Beijing, 100191, P.R. China; E-Mails: pujh@buaa.edu.cn; reimiss@gmail.com; lyunlu@gmail.com; xiongz@buaa.edu.cn

**Keywords:** Wireless Sensor Networks, Coverage Control Protocol, Perimeter Coverage, Off-duty Eligibility Rule

## Abstract

In a randomly deployed and large scale wireless sensor network, coverage-redundant nodes consume much unnecessary energy. As a result, turning off these redundant nodes can prolong the network lifetime, while maintaining the degree of sensing coverage with a limited number of on-duty nodes. None of the off-duty eligibility rules in the literature, however, are sufficient and necessary conditions for eligible nodes. Hence redundancy or blind points might be incurred. In this paper we propose a complete Eligibility Rule based on Perimeter Coverage (ERPC) for a node to determine its eligibility for sleeping. ERPC has a computational complexity of *O*(*N*^2^log(*N*)), lower than the eligibility rule in the Coverage Control Protocol (CCP), *O*(*N*^3^), where *N* is the number of neighboring nodes. We then present a Coverage Preserving Protocol (CPP) to schedule the work state of eligible nodes. The main advantage of CPP over the Ottawa protocol lies in its ability to configure the network to any specific coverage degree, while the Ottawa protocol does not support different coverage configuration. Moreover, as a localized protocol, CPP has better adaptability to dynamic topologies than centralized protocols. Simulation results indicate that CPP can preserve network coverage with fewer active nodes than the Ottawa protocol. In addition, CPP is capable of identifying all the eligible nodes exactly while the CCP protocol might result in blind points due to error decisions. Quantitative analysis and experiments demonstrate that CPP can extend the network lifetime significantly while maintaining a given coverage degree.

## 1. Introduction

Wireless Sensor Networks (WSNs) hold the promise of many new applications in the area of environment surveillance and target tracking. In such applications, the user is interested only in the occurrence of a certain event, such as target appearances or status changes. Due to the random distribution or mobility of the targets, a certain level of sensing coverage over the field of interest should be maintained to guarantee that events of interest will be captured with minimal delay. The sensing area of a sensor node is often assumed to be a disk bound by a sensing circle of fixed radius *r* centered at the node. The field is said to be *k*-covered or have a coverage degree of *k* if any point contained in it is within the sensing area of at least *k* sensors [1]. In general, coverage degree can be considered as a measure of quality of service (QoS) of a wireless sensor network [2]. The higher the coverage degree is, the better the field is monitored. However, the constrained power supply of sensors cannot justify the scheme in which all sensors are put on duty to achieve a high coverage degree. Continuous working leads to the quick depletion of battery power and this shortens the overall network lifetime. Moreover, sensors have limited processing ability and storage capacity due to low cost and small size [3]. Therefore, power-efficient and lightweight designs to prolong network lifetime without sacrificing the coverage degree are one of the fundamental concerns for wireless sensor networks.

In WSNs, unattended deployment usually causes asymmetric node density in the field. In some sub-areas of the field, the sensing areas of neighboring nodes might overlap with each other, which results in coverage redundancy. This redundancy can be exploited to design energy-efficient coverage control protocols [4-10]. In a *k*-covered field, a node is said to be redundant if each point within its sensing area is already *k*-covered by other active nodes [5]. The main mechanism of the coverage control protocols is to turn off the redundant nodes, which are also called eligible nodes to sleep. Since the coverage degree is maintained by the other on-duty nodes, unnecessary power consumption of eligible nodes is saved to a significant extent. An off-duty eligibility rule to identify eligible nodes is critical to the accuracy and efficiency of coverage control protocols. The two most well-known protocols in literature, the Ottawa protocol [4] and CCP protocol [5], adopt either unnecessary or insufficient rules and as a result, redundancy still exists in the Ottawa protocol and blind points might exist with the CCP protocol. Moreover, the centralized algorithms proposed in [9] and [10] can incur expensive communication overhead in a large scale wireless sensor network, due to information exchange. Given the multi-hop and unattended deployment of wireless sensor networks, a localized protocol is more adaptive to large and dynamic network topology which is expected to be quite frequent in mobile and ubiquitous scenarios.

In this paper, we propose a sufficient and necessary condition for a redundant node, Eligibility Rule based on Perimeter Coverage (ERPC). The concept of perimeter coverage was first proposed in [11] to determine whether a field is *k*-covered by sensor networks. Perimeter coverage provides an efficient approach to the complicated coverage problem by simple geometrical calculation. Based on ERPC, a localized Coverage Preserving Protocol (CPP) is presented to maintain network coverage by scheduling the sleep and active states of eligible nodes. Here we summarize the advantages of CPP over previous studies, i.e., the main contribution of this paper as follows.

1. Since our ERPC is a complete condition to determine an eligible node, the ERPC-based CPP not only eliminates the coverage redundancy completely, but also identifies all the eligible nodes exactly. Therefore, CPP can maximize network lifetime without sacrificing system QoS.
2. Based merely on local information, CPP is more cost-effective, especially in large scale and multi-hop networks, than the centralized protocols described in [9-10]. Although [11] presented a power saving scheme (we denote it by PSS) as a possible extension to the perimeter coverage problem, PSS requires much more information exchange and computation time than our work.
3. CPP is capable of maintaining the network to the specific coverage degree requested by an application, while the Ottawa protocol does not support a configurable coverage degree.
4. The computational complexity of ERPC is *O*(*N*^2^log(*N*)), where *N* is the number of neighboring nodes. Comparing with CCP whose eligibility rule has a complexity of *O*(*N*^3^), CPP is a more lightweight protocol and more suitable for sensors whose computation and storage capabilities are harshly constrained.

The rest of this paper is organized as follows. Section 2 surveys the related work in literature. In Section 3, we describe the network model and problem formulation. Section 4 proposes our method to identify an eligible node and clarifies our advantages over the eligibility rule proposed by [11]. Section 5 introduces our coverage control protocol. In Section 6, we present the simulation results. Section 7 concludes this paper.

## 2. Related Work

The most discussed coverage problems in the literature can be classified into two categories: barrier coverage and full coverage. The barrier coverage problem aims to minimize the probability of undetected intrusion through the barrier formed by sensor networks. There has been substantial research on the barrier coverage problem, for example, in [2, 12-15]. In [2] one kind of barrier coverage problem is addressed to determine the least and most covered paths by which an intruder moves through a field given a set of the initial and final locations. Another kind of barrier coverage is introduced in [13] to determine a path with minimal exposure which reflects the time for a sensor to detect a target. Unlike the rectangular or circular field studied in the prior work, the barrier coverage problem in a thin belt field is extensively researched in [12, 14-15].

In this paper, we focus on another type of coverage problem, the so-called full coverage. Full coverage provides the QoS of minimizing the probability of undetected events in the full range of the field. Instrumented with full coverage, the sensor network is vigilant to capture any interested events which take place any time and anywhere. To minimize the power consumption and deployment cost, one kind of energy-efficient full coverage problem is to derive critical conditions for *k*-coverage. In [16], the authors address the problem of determining the relationship among network parameters to guarantee that the probability of *k*-covered approaches 1 as the number of deployed sensors approaches infinity. A mathematical model is proposed in [17] to calculate the minimal number of sensors needed to reach *k*-coverage given the ratio of the sensing range to the range of the field. In [11], the authors suggest that, given a set of sensors, the whole area is *k*-covered if and only if the perimeter of each sensor' sensing area is covered by at least *k* neighboring sensors. All these research efforts indicate that *k*-coverage can be preserved with only a minimal number of deployed sensors. In fact, due to unattended deployment and physical frangibility, more sensors than this minimal number must be deployed, therefore, turning off some redundant sensors can prolong the network lifetime.

Many energy-efficient protocols have been proposed to ensure a desired node density by exploiting deployment redundancy. In [7], a Geographical Adaptive Fidelity (GAF) algorithm is proposed to reduce overall energy consumption, while maintaining a constant level of routing fidelity. A probing-based density control algorithm called PEAS is proposed in [6] to ensure prolonged network lifetime and sensing coverage. Some functional nodes in PEAS continue working until they drain down the battery energy or fail physically, which might reduce network connectivity. In order to balance energy consumption among the network, the ALUL protocol is presented in [8]. None of all the works above, however, derive complete conditions for redundant nodes for coverage. In fact, their main purpose is to maintain network connectivity, which in most cases does not guarantee coverage.

In [9] and [10], the authors propose coverage control algorithms to extend network lifetime for target tracking sensor networks. The algorithms aim to divide the sensor nodes into a maximum number of disjoint sets, each of which can completely cover all the targets. By activating these sets successively, unnecessary energy can be saved to a maximum extent. The authors prove that determining sum maximum sets is an NP-complete problem. Two heuristic algorithms are presented to approximately address the problem. The major limitation of the centralized algorithms, however, is that heavy communication overhead is introduced due to much information exchange, especially in a mobile and multi-hop sensor network. Hence, there is a strong need to develop localized protocols while preserving the desired coverage.

Localized protocols have recently been presented to provide coverage control while maintaining network longevity. One of the most representative protocols is Optimal Geographical Density Control (OGDC) in [18]. OGDC first computes the position where each active node should locate if a full coverage is achieved. Then OGDC picks the nodes closest to these positions-should-be as active node set and put all the other nodes into sleep to conserve energy. This optimal approach by OGDC is built under an assumption that the network density is high enough that a node can be found at any desirable position. Moreover, OGDC assumes that the field is large enough that the nodes near the boundary of the field can be ignored. However, the two assumptions do not necessarily hold true for most sensor networks. Hence, this paper will discuss the localized coverage control problem while relaxing the assumptions about network density and field range.

**Figure 1.**
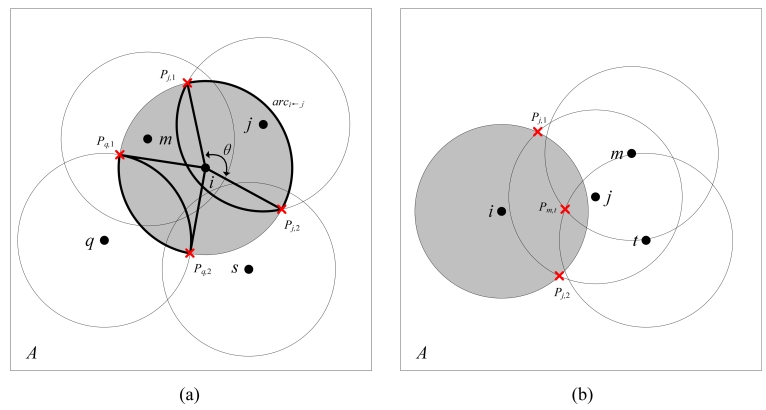
(a) Unnecessary condition of of Ottawa. (b) Insufficient condition of CCP.

The works most relevant to our approach are the Ottawa protocol in [4] and CCP in [5]. Their main approaches are to derive off-duty eligibility rules for redundant nodes and then schedule the work status of these eligible nodes. The off-duty eligibility rule for a sensor to determine whether it is redundant is critical to such protocols. The Ottawa protocol uses a sector to approximately calculate node *i*'s sensing area covered by node *j* as illustrated in Figure 1(a). The sector corresponds to the angle of *θ* and is bounded by radius *iP_j_,*_1_, *iP_j_,*_2_ and *arc_i_*_←_*_j_*. In the eligibility rule of Ottawa protocol, node *i* is said to be eligible for turning off if the sum of the angles created by all of its neighboring nodes are larger than 2π. However, this rule only takes the neighbors within a node's sensing area into account, bypassing the nodes outside the sensing area but still contributing to coverage sponsorship. In the scenario shown in Figure 1(a), the eligible node *i* is considered ineligible by the Ottawa protocol since nodes *q* and *s* are ignored. Therefore, as a sufficient but unnecessary condition, the Ottawa protocol can result in redundancy after turning off only a subset of eligible nodes. An extension to the Ottawa protocol is proposed as the Optimal Coverage-Preserving Scheme (OCoPS) in [19] to provide more accurate coverage control. However, both Ottawa protocol and OCoPS only support 1-coverage and can not meet the requirements of some applications such as target localization or tracking which requires at least 3-coverage[20].

In CCP, a coverage-configurable off-duty rule is adopted to determine node eligibility. The CCP rule considers a node to be eligible if all the intersection points inside its sensing area are *k*-covered. An intersection point is defined as the intersection point of the sensing circles of two nodes or that of the sensing circle of one node with the boundary of the field. The CCP protocol outperforms the Ottawa protocol in coverage efficiency. In the CCP rule, however, the rule does not test the intersection points on a node's sensing circle. As shown in Figure 1(b), the CCP considers node *i* eligible mistakenly based on the assumption that all the inner intersection (i.e. *P_m,t_*) is covered by node *j*. Therefore, the CCP rule is a necessary but insufficient condition for an eligible node and blind points might be incurred, which is verified in our experiment. In this paper, we extend the perimeter coverage lemma in [11] to propose a sufficient and necessary condition for eligible nodes. Based on this complete eligibility rule, our CPP protocol exhibits higher efficiency and accuracy than the two counterparts, the Ottawa and CCP protocols. Moreover, the complexity of our eligibility rule is *O*(*N*^2^log(*N*)), lower than that of CCP rule, *O*(*N*^3^), where *N* is the total number of neighboring nodes. To validate the analysis above, we will integrate the Ottawa and CCP protocols in our simulation and provide a comparative study.

## 3. Network Model and Problem Description

### 3.1. Network Model and Basic Definitions

Consider a square field *A* with side length *L*. Although the field is assumed square in our discussion and simulation, our approach can work in rectangular and circular fields too. We are given a set of sensor nodes, *S*={*i*|1,2,…,*N*} and each node *i* (*i*∈*S*) is located at a known coordinate (*x_i_,y_i_*) inside *A*. Each node has a fixed sensing range of *r*. Moreover, no two nodes are located in the same position.

To better state the coverage problem, we give some basic definitions as follows.

#### Definition 1

The sensing area of node *i* (*i*∈*S*) is defined as a set of points: *D*(*i*)={*p*(*x,y*)|*p*(*x,y*)∈*A* ∧ (*x-x_i_*)^2^+(*y-y_i_*)^2^≤*r*^2^}.

#### Definition 2

A location point *p*(*x,y*) (*p*(*x,y*)∈*A*) is said to be covered by node *i* (*i*∈*S*) if (*x-x_i_*)^2^+(*y-y_i_*)^2^≤*r*^2^ is true.

#### Definition 3

A location point *p*(*x,y*) (*p*(*x,y*)∈*A*) is said to be *k*-covered or have a coverage degree of *k* if it is covered by at least *k* nodes in *S*.

#### Definition 4

The field *A* is said to be *k*-covered or have a coverage degree of *k* if for any location point *p*(*x,y*) (∀*p*(*x,y*)∈*A*), its coverage degree is no lower than *k*. For a specific application, the sensor network is expected to achieve a given coverage degree which is defined as the requested coverage degree.

#### Definition 5

For a sensor network with a requested coverage degree of *k*, a location point *p*(*x,y*) (*p*(*x,y*)∈*A*) is said to be a blind point if this point is less than *k*-covered.

Unlike the Ottawa protocol, we consider all the nodes with a distance within 2*r* to a node since all theses nodes contribute to coverage sponsorship.

#### Definition 6

The neighboring nodes of node *i* (*i*∈*S*) are defined as: *N*(*i*)={*j*|*j*∈*S* ∧ (*x_i_-x_j_*)^2^+(*y_i_-y_j_*)^2^≤(2*r*)^2^, *i*≠*j*}.

Moreover, we assume a simple communication model adopted by [5] as follows.

#### Definition 7

For any two nodes *i* and *j* (∀*i, j*∈*S*), they can communicate with each other if and only if (*x_i_-x_j_*)^2^+(*y_i_-y_j_*)^2^≤*R*^2^ is true, where *R* is the communication radius of each node.

In order to minimize energy consumption caused by communication, we employ a communication radius of *R*=2*r* to ensure that only the neighboring nodes can hear each other (The Micaz[21] sensor platform by Crossbow supports adjustable transmission control scheme.).

The overlapped sensing areas can result in redundant nodes which are defined in [5] as follows.

#### Definition 8

Node *i* (*i*∈*S*) is said to be a redundant node if and only if each point within its sensing area is at least *k*-covered by other active nodes.

**Figure 2.**
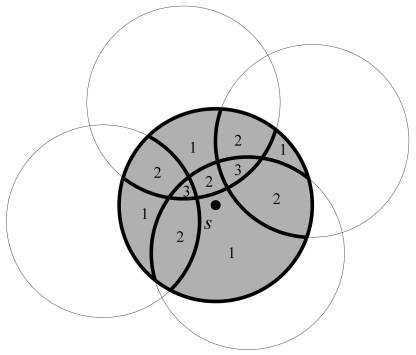
An example of coverage redundancy.

As illustrated in Figure 2, the sensing area of node *i* is completely covered by its neighboring nodes, by Definition 8, node *i* is a redundant node. Turning off redundant nodes can save unnecessary power consumption. Hence, a redundant node is also called an off-duty eligible node.

### 3.2. Problem Description

As shown in Figure 2, one direct solution to determine a redundant node is to find out all sub-regions divided by the sensing circles of all neighboring nodes and check if each sub-region is *k*-covered or not. However, calculating the concave or convex shaped sub-regions might be a highly computation- intensive task for a resource-constrained sensor [11].

Therefore, the energy-efficient coverage problem to be addressed in this paper is formulated as follows:

Given a field *A*(*L*×*L*), a set of sensors *S*, a sensing radius *r* and a requested coverage degree *k*, propose an off-duty eligibility rule for a node *i* (*i*∈*S*) to determine whether it is a redundant node. It is required that such an eligibility rule be a sufficient and necessary condition for an eligible node and can be executed at a low computational complexity. Moreover, for all the eligible nodes identified by ERPC, a sleep scheduling protocol is needed to balance energy consumption among all the nodes in the network.

## 4. Off-duty ERPC Approach

In this section, we describe our novel localized approach to identify redundant nodes, denoted as Eligibility Rule based on Perimeter Coverage (ERPC). Each node runs ERPC locally to compute the coverage degree of each neighbor's sensing circle within the node's sensing area. By checking such information of all the neighboring nodes, the eligibility of a particular node can be determined.

### 4.1. ERPC Theorem

#### Definition 9

The sensing circle of node *i* (*i*∈*S*) is called the perimeter of the node and is defined as a set of points: *P*(*i*)={*p*(*x,y*)|*p*(*x,y*)∈*A* ∧ (*x-x_i_*)^2^+(*y-y_i_*)^2^=*r*^2^}; An arc segment of the perimeter of node *i* is denoted as *arc*(*i*) and called an arc segment of node *i*. Obviously, *arc*(*i*)⊆*P*(*i*) holds.

#### Definition 10

Suppose a point *p*(*x,y*) (*p*(*x,y*)∈*P*(*i*), *i*∈*S*), *p*(*x,y*) is said to be perimeter covered by node *j* (*j*∈*S*) if (*x-x_j_*)^2^+(*y-y_j_*)^2^≤*r*^2^ is true. If *p*(*x,y*) is covered by at least *k* nodes except node *i*, we say that the perimeter coverage degree of *p*(*x,y*) is *k* or *p*(*x,y*) is *k*-perimeter-covered.

#### Definition 11

Node *i* (*i*∈*S*) is said to be *k*-perimeter-covered if for any point *p*(*x,y*) (∀*p*(*x,y*)∈*P*(*i*)), it is perimeter covered by at least *k* nodes other than node *i*. Similarly, an arc segment of node *i, arc*(*i*) (*arc*(*i*)⊆*P*(*i*)), is said to be *k*-perimeter-covered if for any point *p*(*x,y*) (∀*p*(*x,y*)∈ *arc*(*i*)), its perimeter-coverage is no smaller than *k*.

A perimeter coverage lemma is proposed in [11] to determine whether a field *A* instrumented with a sensor network *S* is sufficiently *k*-covered. They can be stated as follows.

#### Lemma 1[11]

Any arc segment of node i' (i∈S) sensing circle divides two sub-regions in the field A. If this arc segment is k-perimeter-covered, the sub-region that is outside node i's sensing area is k-covered and the sub-region that is inside node i's sensing area (k+1)-covered.

Based on Lemma 1, we can obtain the following lemma.

#### Lemma 2

In a sensor network S with a requested coverage degree k, node i (i∈S) is a redundant node if and only if any neighboring node j (j∈N(i)) is k-perimeter-covered when node i is ignored.

#### Proof

For the “if” part, each sub-region inside the sensing area of node *i* is bounded by at least one arc segment of a neighbor's sensing circle. Since the perimeter coverage degree of each neighboring node is still *k* after the removal of node *i*, by Lemma 1, each sub-region inside the sensing area of node *i* is either *k*-covered or (*k*+1)-covered, which means the sensing area of the absent node *i* is sufficiently compensated by its neighboring nodes. Hence proves the “if” part.

For the “only if” part, we prove by contradiction. Suppose there exists a neighboring node *m* (*m*∈*N*(*i*)) whose perimeter coverage degree drops from *k* to *k_m_* (*k_m_*<*k*) due to node *i*'s absence. Consider the arc segment of node *m*'s sensing circle within node *i*'s sensing area. There are two sub-regions divided by this arc segment. According to Lemma 1, the sub-region outside the sensing area of node *m* but inside the sensing area of node *i* is *k_m_*-covered. This contradicts the assertion that any point in the sensing area of node *i* is *k*-covered by its neighboring nodes since node *i* is redundant, hence proved.

Lemma 2 justifies that a node is redundant if and only if no neighboring node is less than *k*-perimeter-covered due to its absence. However, using Lemma 2 as an eligibility rule requires collecting the perimeter coverage degrees from all the neighboring nodes, which can result in much message exchange among nodes. From Lemmas 1 and 2, we derive a more localized approach called Eligibility Rule based on Perimeter Coverage (ERPC) as follows.

#### Theorem 1

In a sensor network S with a requested coverage degree k, node i (i∈S) is said to be eligible for turning off if and only if for each neighboring node j (j∈N(i)) of node i, the arc segment of node j's sensing circle within the sensing area of node i is k-perimeter-covered other than node i.

#### Proof

The proof is directly from Lemma 2. For the “if” part, since the arc segment of each neighboring node *j*'s sensing circle within the sensing area of node *i* is still *k*-perimeter-covered when excluding node *i*, the perimeter coverage of each node *j* remains *k*. By Lemma 2, node *i* is an eligible node, which proves the “if” part. For the “only if” part, we prove by contradiction. Let node *m* (*m*∈*N*(*i*)) be the neighboring node whose arc segment covered by node *i* is *k_m_*-perimeter-covered (*k_m_*<*k*) after node *i* is turned off. Since node *i* is redundant, all its neighboring nodes are *k*-perimeter-covered, which is contradictory with the assertion that there exists a neighboring node *m* that is *k_m_*-perimeter-covered. Hence proves the “only if” part.

**Figure 3.**
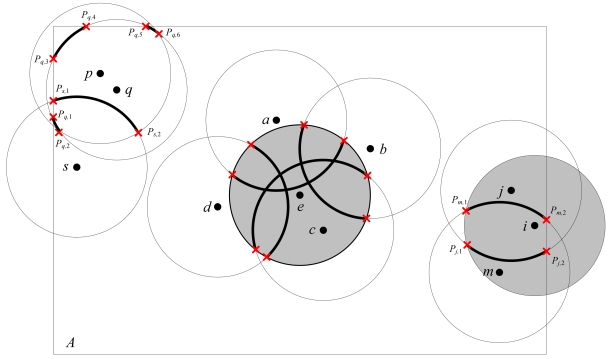
Examples of ERPC.

For the case in which some nodes' sensing areas may exceed the boundary of the field *A*, ERPC also holds since the sensing area of a node is defined as the intersection area of the disk area of the node and the field *A* in Definition 1. As illustrated in Figure 3, node *e* and *i* are identified as eligible nodes for 1-coverage by ERPC. The node *p* is not eligible since the arc segments [*P_q_*_,3_, *P_q_*_,4_] and [*P_q_*_,5_, *P_q_*_,6_] within node *p*'s sensing area are not perimeter covered by any nodes other than node *p*.

By running ERPC locally, a node can identify its off-duty eligibility only based on the location information of its neighboring nodes. Therefore, ERPC is a localized approach and incurs limited communication overhead to the network.

### 4.2. Differences between ERPC and [11]

In [11], Huang *et al.* proposed the use of perimeter coverage to address the problem of how to determine if an area is sufficiently *k*-covered by a given sensor network. According to their solution, the overall system cost can be minimized significantly by periodically examining the network coverage during the deployment process. For a senor network already redundantly deployed, however, [11] does not derive an eligibility rule by which eligible nodes can be identified and then turned off to avoid unnecessary energy drain. The authors of [11] pointed out that a power saving scheme (PSS) might be an extension of perimeter coverage to schedule the sleep and wakeup of nodes. PSS adopts an eligibility rule derived straightforwardly from Lema1[11]:

In a sensor network S with a requested coverage degree k, node i (i∈S) is said to be eligible for turning off if and only if each neighboring node j (j∈N(i)) of node i is still k-perimeter-covered after node i is removed.

However, this eligibility rule, denoted as PSS-ER, does not follow a distributed fashion completely. PSS-ER requires excessive collaborations and communications among neighboring nodes, which is more time-consuming and energy-expensive than ERPC only based on limited local information. The two main advantages of ERPC over PSS-ER are detailed as follows.

1. In each round of PSS-ER, each node has to evaluate its perimeter coverage for two times: the first time evaluation filters out the candidates of eligible nodes; and then, for the second time, each neighbor of any candidate node *i* re-evaluates its perimeter coverage by skipping node *i*. Since the computational complexity of calculating perimeter coverage is as high as *O*(*N*^2^log(*N*)), the two-phase evaluation in PSS-ER takes more time than the one-off judgment used in ERPC.
2. ERPC and PSS-ER share one thing in common at that both of them require information exchange with neighbors when collecting neighbor information and announcing eligibility. Apart from such communication cost, however, each candidate node in PSS-ER has to broadcast its candidacy to all of its neighbors after the first evaluation phase ends. Therefore, PSS-ER incurs much more communication overhead into the network than ERPC does.

In a word, compared with the macro view of the work in [11], ERPC solves the coverage problem from a micro prospective, which identifies an individual redundant node rather than the redundant network as a whole. The issue addressed by ERPC is more significant and challenging for a large-scaled and redundant-deployed sensor network. Moreover, as a completely distributed scheduling scheme, ERPC presents to be more time-saving and power-efficient than PSS-ER proposed by [11], which will be validated in our simulations in Section 6.

### 4.3. ERPC Algorithm

**Figure 4.**
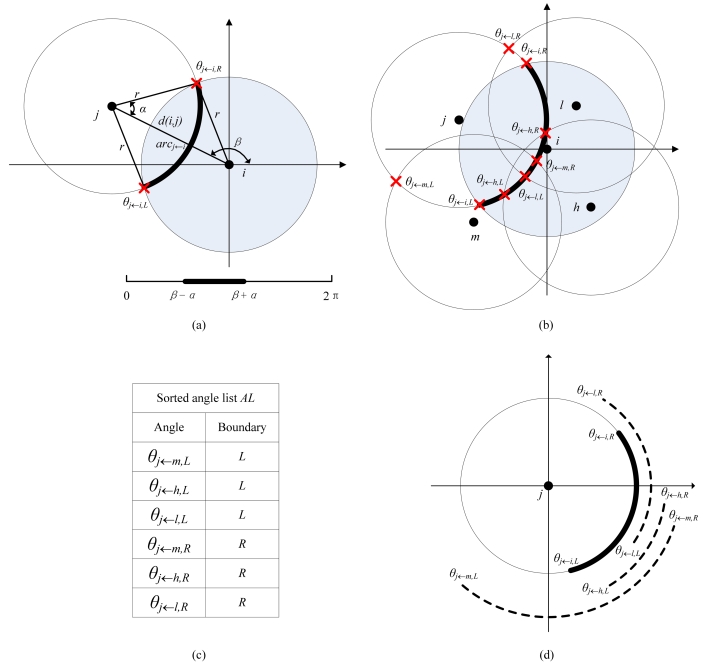
Calculation of the perimeter coverage degree of an arc segment in the ERPC algorithm.

The main part of the ERPC algorithm is to determine the perimeter coverage degree of the arc segment of each neighboring node within a node's sensing area. The whole algorithm of ERPC that runs at node *i* is detailed as follows:

1. For a node *j* (*j*∈*N*(*i*)), let *d*(*i,j*) be the distance between node *i* and *j*. Then, calculate the length of the segment of node *j* covered by node *i*. As shown in Figure 4.(a), the *arc_j←i_* can be measured by its central angle: [*θ_j←i,L_, θ_j←i,R_*]=[*β-α, β*+*α*], where *α*=*arccos*(*d*(*i,j*)/2*r*), *β*=*arctg*((*y_i_-y_j_*)/(*x_i_-x_j_*)).
2. For node *j*'s each neighboring node *m* (*m*∈*N*(*i*)∧*m*≠*i*), calculate node *j*'s arc segment covered by node *m*, denoted by [*θ_j←m,L_, θ_j←m,R_*], as illustrated in Figure 4(b).
3. Add all the points *θ_j←m,L_* and *θ_j←m,R_* generated by last step to an angle list *AL* and then sort AL in an ascending order. Meanwhile, mark each point as a left or right boundary of each covered arc segment, as shown in Figure 4(c).
4. As demonstrated in Figure 4(d), first calculate the perimeter coverage degree of the start point of *arc_j←i_*, denoted as *k_temp_*. Then, scan the arc segment [*θ_j←i,L_, θ_j←i,R_*] by visiting each point in the sorted *AL*: whenever a start point is visited, *k_temp_* is increased by one; whenever an end point is visited, *k_temp_* is decreased by one. Finally, the perimeter coverage degree of *arc_j←i_* should be the minimal value of *k_temp_* during the scanning process.
5. For each node *j* (*j*∈*N*(*i*)), check the perimeter coverage degree of its arc segment within node *i*'s sensing area by running the above 4 steps. If there exists a node whose arc segment covered by node *i* is less than *k*-perimeter-covered, node *i* considers itself ineligible. If no such a node is found, node *i* determines it is eligible.

### 4.4. Complexity Analysis

Consider the algorithm in Section 4.2. In a network with *N* nodes, the maximum number of nodes that are neighboring to a node is *N*. The first four steps of the ERPC algorithm are performed to determine the perimeter coverage degree of a covered arc segment. In the second step, calculating all the arc segments of all the neighbors has a complexity of *O*(*N*). The complexity of the quick sort algorithm in the third step is *O*(*N*log(*N*)). In the fourth step, the scanning process has a complexity of *O*(*N*). Hence, the complexity to calculate an arc's perimeter coverage degree is *O*(*N*log(*N*)). Since the fifth step tests all the *N* neighboring nodes to draw a final decision, the overall complexity for the ERPC algorithm is *O*(*N*^2^log(*N*)). The rule used in the CPP protocol checks whether all the intersection points between nodes' sensing circles are *k*-covered to identify an eligible node. Since the number of the intersection points between *N* nodes is *O*(*N*^2^) and the complexity to calculate the coverage of an intersection point is *O*(*N*), the overall complexity of CCP rule is *O*(*N*^3^). Therefore, ERPC is a more lightweight off-duty eligibility rule than CCP rule.

## 5. Coverage Preserving Protocol

After turning off the eligible nodes filtered out by ERPC, the network coverage degree can be preserved by the remaining active nodes. If these on-duty nodes continuously work, however, they may soon run out of battery energy. This working model might not be desirable since the failure of some functional nodes can result in partitioning of the network or isolation of nodes. In this section, we propose a Coverage Preserving Protocol (CPP) to balance energy consumption among the neighboring nodes while maintaining the requested coverage degree. In CPP, a node can work at one of three states: Sleeping (Off-duty), Active (On-duty) and Listening. The operation of each node is divided into rounds. Each round takes the same period of time (*T_r_*) and consists two steps detailed as follows.

### 5.1. Neighbor Information Collection

At the beginning of each round, all nodes are in On-duty state. To obtain the information of neighboring nodes, each node broadcasts a Beacon Message (BM) which contains node ID and its current location. Then, each node enters Listening state to collect the BMs from its neighbors. Finally, a neighbor list is maintained at each node. Since nodes may have some mobility in some mobile ubiquitous applications, it is necessary for each node to update its neighbor list in each round (we assume that each node can obtain its location information by GPS or other self-localization schemes such as DV-hop[22].)

### 5.2. Back-off based Eligibility Evaluation

After collection of neighbor information, each node evaluates its eligibility by ERPC. However, blind points may occur due to some neighboring nodes' dependency on each other, as shown in [4]. CPP adopts the back-off scheme in [4] to avoid blind points. In this scheme, each node runs ERPC after a random delay timer *T_d_*. The node with the shortest *T_d_* evaluates its eligibility earliest. If a node considers itself eligible by ERPC, it broadcasts a Quit Message (QM) to declare that it enters Sleeping state. The neighboring nodes with longer *T_d_* receive the QM and remove the sleeping node from their neighbor lists. Thus, a node with a longer *T_d_* will evaluate its eligibility without taking the sleeping nodes into account. Furthermore, by the back-off scheme, the candidate nodes that dependent on each other compete to be eligible by rounds in a random fashion, which evenly spreads the energy consumption around all nodes.

**Figure 5.**
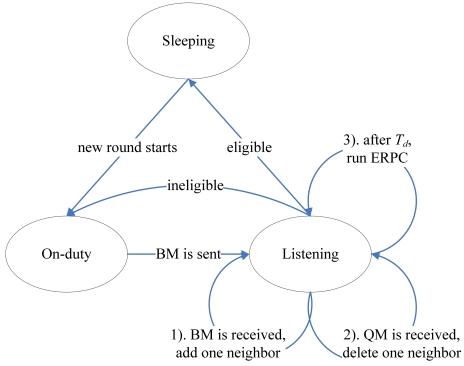
State transition in CPP.

In Sleeping state, the eligible node is turned off to save battery energy. In On-duty state, the node performs the normal sensing and processing tasks. In Listening state, the node 1). first adds one neighbor in case that a BM is received, and then 2). deletes one neighbor upon QM and finally 3). evaluates its eligibility by ERPC after *T_d_*. The state transition in CPP can be illustrated as Figure 5.

### 5.3. Discussion about T_r_ and T_d_

The back-off scheme in CPP employs a randomized delay timer *T_d_* to avoid blind points caused by multiple eligible nodes. When deciding *T_r_*, we mainly consider two factors: message exchange delay and remained energy. First, to bound the delay within a reasonable interval, we suggest that *T_d_* should be a fraction of the sum of the round-trip delays among neighborhood. Therefore, we derive a back-off delay in the form as

(5.1) $$T_{d}=\mathrm{RNDM}\left(0,1\right)\times N_{d}\times T_{rt}$$

where *RNDM*(0,1) represents a random value uniformly distributed between [0,1], *N_d_* indicates node density defined as π*r*^2^*N/L*^2^, and *T_rt_* is the round-trip delay for a QM packet to travel over the wireless link. In a scenario where the wireless bandwidth is 256 kbps and the packet length of QM is 32 bytes, *T_rt_* is typically 2 ms.

Moreover, we take the remained energy at each node into account. Suppose that all nodes have different energy levels at the very beginning. Let *E_r_* denote the amount of energy at a node that remains and *E_m_* denote the amount of initial energy. A fair consideration is to ensure that a node with a lower *E_r_/E_m_* should be more likely eligible for sleep than the node with a larger *E_r_/E_m_*. As a result, the energy consumption can be evenly spread around all the nodes. Therefore, combining with Equation 5.1, the final form of the delay timer can be stated as:

(5.2) $$T_{d}=\left(\frac{E_{r}}{E_{m}}+\mathrm{RNDM}\left(0,1\right)\right)\times N_{d}\times T_{rt}$$

As for the length of each round, it has little impact on the total working time of each node in all rounds. However, frequent round switch would result in much energy drain. Hence, *T_r_*≫*T_i_*,*_d_* is generally enough. In the simulation we choose 100 s as *T_r_* for a 1000-second-long running process if not specified otherwise.

## 6. Performance Evaluation

In this section, we evaluate the performance of CPP in simulation experiments. Two of the best-known protocols, the Ottawa protocol and the CCP protocol, are introduced for comparison. We implement CPP in Matlab 7.0[23]. In the following experiments, the range of the field *A* is 50 m × 50 m if not specified otherwise, and the sensing radius of each node is 10 m. In the graphs of all the experiments, each data point represents the average value of 10 trials with different random nodes distributions.

### 6.1. Achieved Coverage Degree

In this experiment, we compare CPP to the Ottawa protocol in the performance of the achieved network coverage degree which reflects protocol efficiency. To evaluate coverage, we divide the entire field into grids with the size of 1m×1m. The coverage degree of each gird can be measured by checking the number of on-duty nodes that cover the center of the grid. Hence, the achieved coverage degree of the field can be approximately calculated by averaging the coverage degrees of all grids.

**Figure 6.**
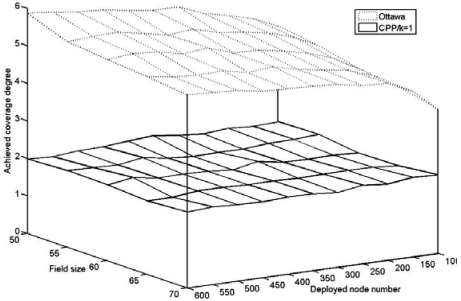
Achieved coverage degree.

Figure 6 illustrates the network coverage degrees achieved by CPP and the Ottawa protocol. The requested coverage degree is *k*=1 in CPP. It can be seen that the achieved coverage degree in CPP keeps around 2 no matter how many the deployed nodes are and how much the field size is. In contrast, the coverage degree achieved by the Ottawa protocol is as high as 5 or 6, and rises as the number of the deployed node increases. This is because that the Ottawa protocol only utilizes information of the nodes within the sensing area, while CPP makes use of all the nodes within twice the sensing range. The eligible nodes identified by the Ottawa protocol are only a subset of all the should-be-eligible nodes in the network. On the contrary, after running CPP, only a minimal number of nodes remain active to preserve the desired coverage degree and all the eligible nodes are turned off to conserve energy. This can be explained by the reason that the adopted ERPC in CPP is a complete approach to determine eligible nodes. Therefore, CPP outperforms the Ottawa protocol in the efficiency of exploiting the coverage redundancy.

### 6.2. Incurred Blind Points

In a surveillance sensor network, full coverage is expected to ensure real-time monitoring of the interested events. Therefore, blind points should be avoided to improve system alertness and reliability. Since the blind points caused by random deployment can not be controlled, this experiment evaluates the number of the blind points incurred only by protocols.

**Figure 7.**
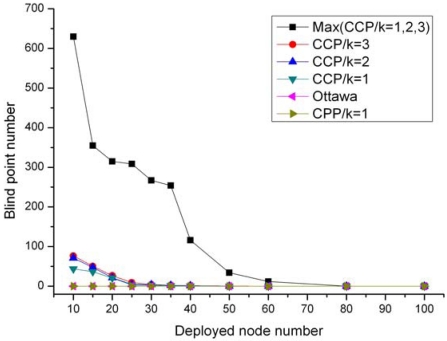
Blind points incurred by protocols.

The number of the blind points caused by the three protocols is demonstrated in Figure 7. The symbolized line marked with “Max(CCP/*k*=1,2,3)” represents the maximal number of the incurred blind points among different requested coverage degrees. The Ottawa protocol and our CPP introduce no blind points to the network, since both of them adopt sufficient rules to determine eligible nodes. In contrary, we observe that CCP may result in blind points at all the three requested coverage degree. Moreover, the maximal number of the blind points caused in CCP is more than 600 especially in a densely deployed sensor network. This happens because CCP ignores checking the coverage of the intersection points on the perimeter of a node's sensing area and hence turns off some ineligible nodes by mistake.

### 6.3. On-duty Node

**Figure 8.**
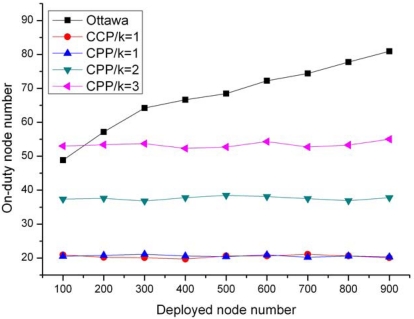
On-duty nodes used by protocols.

Figure 8 compares the number of on-duty node after running the three protocols. It can be observed that, when *k*=1, both of our CPP and the CCP protocol generate the equal number of on-duty node and the number remains around 20 as the deployed nodes increases from 100 to 900. This result indicates that CPP has the equivalent efficiency in maintaining network coverage. Moreover, the number of on-duty node used by CPP increases to about 38 and 53 on average, and keeps steady when *k*=2 and 3, which means CPP only activates the exact nodes that should wakeup.

While comparing to the Ottawa protocol, CPP needs much less active nodes when *k*=1 and the number of the deployed nodes is 100. When the deployed node number reaches 900, the active nodes number in the Ottawa protocol rises to 80, while our CPP remains the same level of on-duty nodes. This occurs because the Ottawa rule evaluates node eligibility only based on the knowledge of a small part of neighbors. Furthermore, in the Ottawa protocol, all the nodes close to the boundary of the field remain active at all the rounds since these nodes are refused to be tested the Ottawa rule directly. In contrast, the ERPC in CPP addresses the boundary problem accurately. Therefore, CPP can preserve the network coverage with fewer on-duty nodes than the Ottawa protocol, which means CPP is a more energy-efficient coverage control protocol.

### 6.4. Coverage Configurability

**Figure 9.**
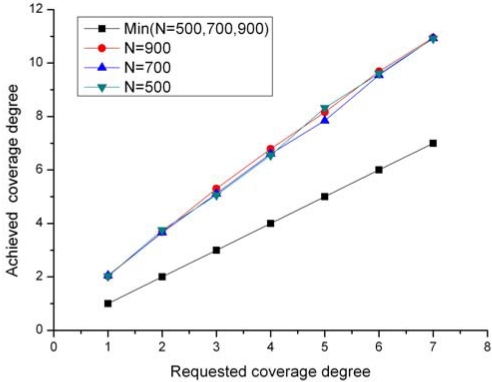
Achieved coverage degree by CPP.

In this experiment we evaluate the ability of CPP to configure the network to the requested coverage degrees. Figure 9 demonstrates the achieved coverage degrees in CPP under different requested coverage degree (*k*=1∼7) and different network scales (*N*=500, 700 and 900). The group of data points labeled “Min(*N*=500, 700, 900)” denotes the minimum achieved coverage degree among all grids for different requested coverage degree. From the results, we can see that the achieved coverage degree in CPP is almost proportional to the requested coverage degree for different numbers of the deployed nodes. This result demonstrates that CPP can scale to any coverage degree requested by a specific application. Moreover, the minimum coverage degree keeps equal to the requested coverage degree. This means CPP does not incur any unnecessary coverage redundancy to the network.

### 6.5. Convergence Time

In this experiment, we evaluate the time taken by each protocol to filter out eligible nodes. At the beginning of each round, all nodes are active and then they perform the specific eligibility rule to decide whether to turn off itself. As this eligibility evaluation proceeds, the number of on-duty nodes decreases until all the exact eligible nodes are identified. We define convergence time (*T_c_*) as the continuous running time in each round before the on-duty nodes reaches a minimum number. Convergence time reflects both computation and communication complexity of coverage control protocols. A shorter convergence time means that the protocol enters the stable state more quickly and has more time to perform surveillance tasks. In addition to the three protocols, we implement PSS proposed by [11] to clarify the differences between PSS and CPP. PSS adopts an eligibility rule based on perimeter coverage that a node is redundant if and only if all of its neighboring nodes are *k*-perimeter-covered when skipping it. We simulated the four protocols under the same scenario in which the total number of deployed nodes is 100 and the requested coverage degree is 1. Moreover, we choose 50s as *T_r_* in a 500-second-long simulation. The experiment results are illustrated in Table 1.

It can be observed that Ottawa has the best convergence time performance. This can be expected since the computational complexity of Ottawa is the lowest among all the protocols and, moreover, each node in Ottawa broadcasts at most twice. At the end of the eligibility evaluation, however, Ottawa results in the most on-duty nodes due to its unnecessary eligibility rule. CCP, PSS and CPP perform closely in the number of on-duty nodes since all of them adopt a necessary eligibility rule. PSS spends the most time in filtering out all the eligible nodes. The reason is that, as discussed in Section 4.2, the 2-phase eligibility evaluation and 3-times broadcasting are time-consuming indeed. Since the proposed CPP reduces the computational complexity to *O*(*N* ^2^log(*N*)), it takes less convergence time than CCP.

### 6.6. Network Lifetime

This experiment evaluates CPP's ability to prolong network lifetime. The metric used in evaluation is the α-coverage lifetime which is defined by [18] as the continuous running time of the network before the ratio of 1-covered area to the total area drops below α.

In this simulation, 100 nodes are randomly deployed in the field of 50 m × 50 m and each of them starts with an initial energy of 200 Joules. In addition, we follow the energy model in [5], where the power consumption of Tx (transmit), Rx (receive and listen), Idle and Sleeping modes are 1400 mW, 1000 mW, 830 mW and 130 mW respectively. Apart from the four coverage control protocols, we also simulate an original network with all nodes on to evaluate how far the network lifetime is improved by scheduling schemes.

**Figure 10.**
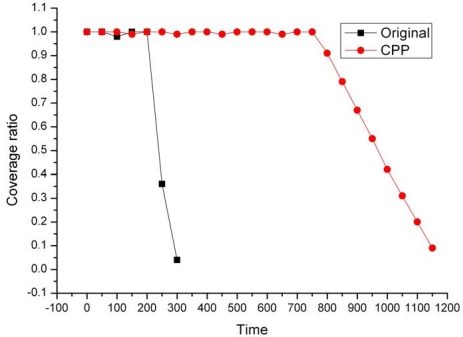
Dynamic coverage ratio.

Figure 10 compares the dynamic coverage ratio of the original network and the CPP network. We sample the coverage ratio from this simulation every 10 seconds. Before the coverage ratio decreases below 90%, CPP can provide more than 4 times of the lifetime of the original network. This can be explained by the fact that CPP filters out the exact nodes to maintain network coverage and as a result, the energy of redundant nodes is significantly conserved.

**Figure 11.**
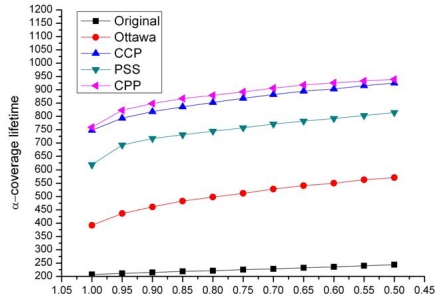
Network α-coverage lifetime.

Figure 11 shows the network α-coverage lifetime resulted by different protocols when varying α from 100% to 50%. As expected, the original network without using any scheduling schemes drains energy most quickly among four protocols. The α-coverage lifetime of such a network holds no longer than 250 s which is roughly the lifetime of an all-time idle node. The Ottawa protocol provides about twice as long as the lifetime of the original network. Due to the redundancy remained in Ottawa network, however, the lifetime of the Ottawa protocol is bounded within 600 s. CCP and CPP present a similar capability in extending network lifetime. Both of them offer a lifetime more than 800s even when α is as high as 90%. This is because the eligibility rules adopted by the two protocols filter out a minimum number of nodes to preserve network coverage. Although CPP requires an excessive number of nodes to avoid blind points, we can observe that the lifetime of our CPP is slightly longer (about 10s) than that of CCP for each α. This is mainly due to two reasons. First, CCP consumes much energy to periodically broadcast HELLO messages. Second, in CCP a node takes more time to execute the *O*(*N*^3^)-complicated eligibility rule and consequently stays awake longer than in CPP. Moreover, we can observe that the network lifetime in CPP rises more than 20% when comparing with the PSS proposed in [11]. This can be explained by the reason that PSS requires one more time broadcasting than CPP, which consumes much more energy.

### 6.7. Evaluation Summary

In summary, we draw the key results from our experiments as shown in Table 2. Our CPP outperforms the Ottawa protocol, both in coverage efficiency and coverage configurability. Meanwhile, CPP and CCP have equivalent performance in the above two metrics. Moreover, as for coverage accuracy, CPP can better ensure network reliability and system alertness than CCP. As for the α-coverage lifetime, CPP has an overwhelming advantage over the other two protocols when α=90%.

## 7. Conclusions

In this paper we investigate the coverage control protocol which reduces energy consumption by turning off redundant nodes. We propose an off-duty eligibility rule, denoted as ERPC, to determine redundant nodes. To the best of our knowledge, ERPC is the first work to provide a sufficient and necessary condition of off-duty eligible nodes. Moreover, ERPC has a lower computational complexity than the most well-known CPP rule. A Coverage Preserving Protocol is developed to schedule the work states of candidate eligible nodes. The localized CPP is more self-adaptive and energy-efficient in a large scale and multi-hop sensor networks. Moreover, CPP supports configurable coverage degree to meet various application requirements. Simulation results indicate that CPP can preserve the network coverage efficiently and accurately. Moreover, CPP can extend the network lifetime up to 4 times without sacrificing system reliability. Most studies including our CPP require that each node knows its own location. To relax such deployment restrictions, we will investigate the possibility of deriving a location-independent coverage control protocol in the future.

## Figures and Tables

**Table 1.** Convergence time.

**Protocol**	**Metric**	**Convergence Time (ms)**	**On-duty Node**	**Computational Complexity**	**Broadcasting Times**
Ottawa		1324	48.2	*O*(*N*)	2
CCP		2578	20.8	*O*(*N*^3^)	2
PSS		3659	21.1	2*O*(*N*^2^log(*N*))	3
Our CPP		2108	21	*O*(*N*^2^log(*N*))	2

**Table 2.** Comparison among protocols.

**Protocol**	**Metric**	**Coverage Efficiency**	**Coverage Accuracy**	**Coverage Configurability**	**90%-Coverage Lifetime (s)**
Ottawa		Much redundancy	No blind points	1-coverage	661
CCP		No redundancy	Blind points	*k*-coverage	818
Our CPP		No redundancy	No blind points	*k*-coverage	848
